# Implications of migration on health and education: returned migrants and school teachers perspective in India: A qualitative study

**DOI:** 10.1016/j.jmh.2024.100289

**Published:** 2024-12-27

**Authors:** Bernard Attah-Otu, Nikita Jaiswal, Priya Gupta, Angan Sengupta

**Affiliations:** a Amrita School for Sustainable Futures, Amrita Vishwa Vidyapeetham, Amritapuri Campus, Kollam, India; b University of New Mexico School of Medicine, USA; c Amrita School of Business, Amrita Vishwa Vidyapeetham, Bangaluru, India; d Amrita School of Business, Amrita Vishwa Vidyapeetham, Bangaluru, Karnataka, India

**Keywords:** Migration, Labour migration, Health, Education, Left-behind, Accompanied children, Tribal communities, India

## Abstract

**Background:**

Poor tribal communities migrate from rural to urban areas to tackle their financial hardships. However, limited empirical literature collectively delves into the implications of health and educational attainments of household members of migrant households, while examining the intricate dynamics of labour migration among tribal Indian communities in India.

**Objective:**

This study aims to examine the effects of labour migration on household health and education for accompanied and left-behind children.

**Methods:**

Twenty-two semi-structured interviews and two focus group discussions (6–8 participants) were conducted in four tribal communities in India from August to November 2022 comprising 14 migrant households that recently returned from migration, three left behind adolescents of school age, three primary school head teachers and two Panchayat heads (community head). Data were analysed through an inductive thematic approach.

**Results:**

Participants identified a lack of adequate housing and sanitation conditions, with intense and long labour hours as conditions at migration destinations that affect their health. Lack of maternal and child healthcare is staggering at the place of work. In particular, our findings revealed that temporary labour migrants do not enrol their children in schools; however, the case was different from permanent and long-term migrants who enrol their children in private schools. At their place of origin, school teachers reported frequent school dropouts, low enrolment, attendance and poor academic achievements among children of migrating households. There is prominent gender dynamics in academic performances, and its determinants. Early age marriage and joining labour force dropping-out of school is common.

**Conclusion:**

The results highlight the negative effects of migration as a livelihood strategy on rural communities, particularly in the health and education of household members, asking for immediate government interventions.

## Highlights

• Migrants often experience an improvement in socioeconomic status through remittances.
• Challenges at migration destinations include inadequate housing, sanitation facilities, and limited healthcare access.
• Grandparents and guardians are not able to adequately supervise the education and health practice of left-behind children.
• Low educational development in tribal communities due to frequent dropouts by children of school age for migration.
• Left behind take part in household chores and economic activities that affect their school enrolment and attendance.

## 1 **B**ackground

Migration plays a key role in the lives of rural communities, serving as adaptation and mitigation strategies for better livelihood (De Haan, 2002; Sarawary et al., 2021; Viswanathan and Kumar 2015). It is also widely recognised that migration has a positive effect on the economy and the migrant household (George et al., 2011;), through remittances, the acquisition and transfer of skills, and human capital development. Besides these positive effects, there are several factors of labour migration that adversely affect the migrant or their family that are hardly discussed on a large scale. Furthermore, migration destinations are not able to offer the same economic opportunities in the form of employment for all migrant populations due to surplus labour in urban areas. Because of inadequate employment opportunities and precarious living conditions at the destination, internal labour migrant households and the left-behind family members, especially women and children, find themselves in a vulnerable situation due to a shortage in expected or projected migration benefits. These constraints of the absence of suitable employment opportunities and precarious lives in urban areas have over time become obstacles that hinder the migrants' household aspirations and capability to function effectively (De Haas, 2021), especially for migrants within developing countries.

In India, the internal migration rate is staggering and huge (Rameshan et al., 2019; Srivastava, 2020) with frequent interstate and inter-district short-term labour migration. India's internal migration is characterised by the mass movement of the entire family from the place of origin (Betancourt, et al., 2013) to the urban centres for labour employment. However, in urban areas, labour migrants' expectations of a quality life and income opportunities are often unmet. Many migrants find themselves in precarious employment, informal jobs, and vulnerable health-threatening situations (Srivastava, 2020). They often find themselves living in slum conditions (Dhal et al., 2020), and with limited education opportunities for accompanied children of school age. The left-behind household members also suffer due to the absence of adequate remittance from the migrating household members.

The effects of migration cuts across age, gender, and among migrating and left-behind household members. Previous empirical research on the effects of migration has shown that migration affects both migrating household members and those left behind (Coffey, 2013; Heller and Kaushik, 2020; Hu, 2012; Roy et al., 2015). Mckenzi and Rapport (2011) recorded a reduction in attendance and education attainment among children in migrating households in Mexico because of the frequent dropout rates. In China, Hu (2012) recorded a negative effect of migration on children's education within households with frequent absences of adults. The study also noted more severe effects on girls and in households that face regular financial. constraints.

Furthermore, studies in India have examined and documented the effects of migration on migrating and left-behind family members (Green et al., 2019; Jain and Jayaram, 2023; Roy et al., 2015). Other studies on migration focused on the effects on left-behind wives and women (Kakati, 2014; Lei and Desai, 2021). Additionally, other research studies focused on the nutritional needs of accompanied children (Lei et al., 2020; Kambara et al., 2021; Ravindranath et al., 2019; Vikram, 2018) including the immunisation status of migrant children (Geddam et al., 2018; Kusuma et al., 2010), and the effects of migration on education of children (Roy et al., 2015; Kambara et al., 2021). Despite this extensive literature on the effects of migration on health and education, less attention has been focused on migration conditions that hinder the health and education of children and how migration influences education attainment and enrolment at the place of origin. Only a few limited studies (Kambara et al., 2021; Roy et al., 2015) have focused on the migration effects on education in India. However, these studies did not explore how migration conditions affect the migrant's household health or children's school enrolment and attendance at the place of origin. To the best of our knowledge, only one study (Antia et al., 2022) has explored school teachers' experience and perception of the effects of migration at their place of origin in Georgia.

This study seeks to examine how migration conditions affect the health of migrating household members and the educational attainment of left-behind children of school age This study delves into the lived experience of return migrants at their place of origin, who belong to a tribal community. Most studies on the effect of migration have been conducted at destination places without exploring the socio-economic conditions that motivate the migration process and decisions, and the potential effects on health and education of migrating and non-migrating household members.

Our study's contribution to the literature is threefold: first, we contribute to the existing literature by examining the effect of household migration motivation; second, we explicitly identify migration conditions that determine the health conditions of migrant household members; and third, it is the first attempt to discuss migration scenarios and their effects on education enrolment and attainment

## 2 Theoretical background

The migration and health nexus is a complex one, an association that can be understood through multiple approaches or theories. Among migrants, health status is influenced and shaped by different inherent and external factors within their immediate environment at origin and destination places. Hence, ‘migration’ can be considered as a social determinant of health (Castañeda et al., 2015). Towards a deeper understanding this study has adopted the Social Determinants of Health (SDH) framework as its theoretical framework (Solar and Irwin, 2010**)**. The World Health Organization's Global Commission on Social Determinants of Health (CSDH, 2008) defined social determinants of health (SDH) as “the structural determinants and conditions of daily life” which are “responsible for a major part of health inequities between and within countries.” The SDH framework argues that a person's individual health risk and vulnerability to disease are influenced by different factors such as where the person is born, where the person grows, where the person lives, and where the person works, beside his/her demographic and socio-economic profile. Since, this study focuses on socioeconomically underprivileged tribal migrants, their health is adversely affected by the precarious workplace and housing environment at the place of migration.

## 3 Methods

### 3.1 Study design and setting

This qualitative study analyses ethnographic data from the in-depth interviews involving returned migrant households and left-behind adolescents, as well as key informants (primary school teachers and panchayat leaders) from four tribal villages in Madhya Pradesh, India.

**Fig. 1:**
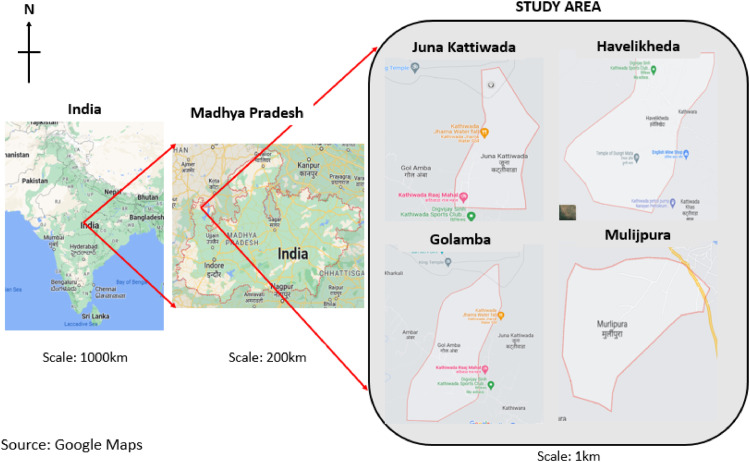
Map of the study location showing the location of the study villages.

The data collection was conducted in Juna Kattiwada (population: 276), Golamba (population: 638), Mulijpura (population: 178), and Havelikheda (population: 698) in the Kattiwada Block of Alirajpur District, Madhya Pradesh, India (Fig. 1).

### 3.2 Participants' recruitment

Participants were recruited from all four villages using the snowball approach, a technique utilized in exploratory studies to reach participants in areas, like tribal communities, where access may be challenging (Atkinson and Flint, 2001). Adolescent participants were identified from the communities during a visit to the middle school with the assistance of the head teacher. Where they expressed their willingness to participate in the discussion. Interviews were conducted in their respective households rather than in the school with the permission of their parents or guardian Mothers for the two girl participants (both aged 17 years, standard 11), and adult siblings for the boy participant (age17, standard 12) were present during the interviews. To ensure that their individual perceptions were adequately captured, the interview guide and questions were kept simple. We used an interactive approach, and the fact that the interviews were conducted in their homes and in the presence of parents or guardians helped ensure that the children were relaxed and comfortable. A consent form, translated into Hindi, was read to each participant, detailing the research's purpose, and all respondents provided verbal consent before interviews, later confirmed with their signatures.

### 3.3 Data collection

A total of twenty-two semi-structured interviews and two focus groups (6–8 participants) discussion were conducted for this study. The semi-structured interviews comprise 14 in-depth interviews (IDIs) with returned migrants, three left-behind adolescents and five key informants interviews (KIIs) with three primary school teachers from three villages and two Panchayat leaders serving as the local administrate heads in the study area. Data were collected from August to November 2022 using an interview guide prepared by the research team. The data collection continued until saturation was reached. We decided that saturation was reached when we have received repetitive information from participants and no new insights emerged during discussion (Braun and Clarke, 2006).

Following the personal interviews, two focus group discussion were held separately for women and men, that helped to avoid issues of dominance or imbalance during discussion. Participants for the FGD were purposefully selected involving household heads or individuals who has not migrated during the last 2–3 years in order to better understand the influence of migration after a considerable time since their migration. Topics discussed during the interview include migration motivation and expectations, migration conditions, including working and accommodation conditions that might affect their health, economic activities, and education status of accompanied children and left-behind children of school age, household members’ health, and the effects of remittances on the household. We also discuss the key factors influencing education scenarios in communities and how migration influences school enrolment and attendance. All Interviews were conducted in Hindi with the assistance of a professional native Hindi speaker. Each set of interviews was audio recorded to ensure that all descriptive narratives were recorded in the field to prevent loss of data.

### 3.4 Data analysis

Two professional native Hindi speakers translated all transcripts verbatim from Hindi to English language studied for a detailed comprehension of information. Each set transcript was analysed independently using Dedoose statistical software vision 9.0.17 and coded thematically (Braun and Clark, 2006) through the iterative interpretive approach (Neale, 2016). Data analysis continue with emergence similar patterns and themes, indicating themes saturation. However, we continue coding of all the transcripts despite saturation until all the interviewed transcripts were completely coded. The first author guided by the research question identified preliminary open and axial codes. A codebook was utilised to verify and redefine initial codes generated through a series of meetings by the research team. Finally, similar codes were categorised, and grouped into themes after a series of verifications by the entire research team. Three meetings took place with the research team to discuss the emerging themes to find patterns and themes emerging from the codes. Throughout the meetings, our interests were deployed towards developing codes and themes that focus on or related to migration condition experiences, education, left behind and teachers' perceived migration experience in the study area. Similar codes and patterns were identified and categorised into themes (Table 1)

**Table 1:** Themes emerging from data analysis.

Key themes and categorise	Sub-themes within themes
Migration decision-making and motivation	Family obligation
	Income generation
	Livelihood Strategies
	Limited opportunities
Migrants' perception of migration	Remittance
Effects of migration on health	Lack of adequate housing
	Absence of sanitation facilities
	Work-related stress
	Absence of health facilities
Effects of migration on children	Accompanied children
	Left-behind children
Effects of migration on education: School teachers' perception	School enrolment and attendance
	Academic performance and graduation rates
	School attendance and family socioeconomic status
	Parents' support and perception of education
	Teachers as tutors and support aids

### 3.5 Ethical approval

The research protocol for this study was approved by the Institute of Medical Science, Healthcare, Education and Research, Institutional Ethics Committee. The ethical protocol submitted for approval includes research instruments and an informed consent form in English and translated into Hindi language barriers. Participation in this study was voluntary. All respondents gave verbal consent and signed a copy of the consent form in Hindi and English.

## 4 Results

Table I presents five main themes and corresponding sub-themes that emerged from the analysis of 22 semi-structured interviews and two focus group discussions.

### 4.1 Migration decision-making and motivation

Migration decisions are typically collective and involve the entire household, considering the expected support for the household.

Both in-depth interviews and focus group discussions (FGDs) consistently highlighted the pursuit of increased income and employment opportunities, the fulfilment of family obligations, and the recognition of limited prospects within their local communities.

“When you have a family and they depend on you to take care of them and you are not able to do so because you do not have land to cultivate crops for them to feed on, you have to migrate to earn money for them.” (Surya, IDI 3)

“Many families do not have land to farm or a tractor that they can hire out, so they are not able to grow crops; they are not able to make money here in the village, so they migrate to earn money for themselves.” (Men FGD)

“People here are poor and do not have land to work (farm), so they move out and migrate to Gujarat because there is no alternative work or other work here in the village, so they have to migrate; it is not a choice; the conditions make them migrate.” (Women FGD)

Participants disclosed household migration decision is influenced by gender and age, with the choice of migration destinations primarily based on job availability. The age at which individuals migrate varies between boys and girls in the communities and across households. Average migration age varies across households and depends on the nature of work available at the migration destination. In general, migration is not encouraged within the community, but rather induced by socio-economic conditions and the financial needs of households, driven by a desire to meet their financial obligations and settle their debts.

“People begin migrating at the age of 15, while the migrating age ranges from 15 to 30 years. Anyone <15 years old can migrate but is not allowed to work in the plantation field or on construction work.” (Panchayat Leader, KII 1)

“There is no restriction on migrating age among the children. If the child is 10–12 years of age, they go with their parents for farm work in Gujarat.” (FGD with men)

“Parents do not encourage their children to migrate. Both husband and wife migrate together to meet the expenses of the family, as the expenses in commodities are very high and kids also have some or the other sort of expenses that must be met by the parents.” (Sudhir, IDI 13)

### 4.2 Perception of migration

Participants shared that their primary reason for migrating is to improve their economic prospects and increase their income. Migration is seen as the easiest means to increase the household as well as community earnings, given the limited opportunities available within the community.

“Migration is improving our lives here in the village. If we go to work and get higher daily wage, it is good for us. When a person stays in the village, they are not able to work or make money, so migrating helps us to get the money that would not have been possible if we had stayed in the village.” (Kannan, IDI 8)

“Migration is a good thing for me, I go outside the village to work, and get good money there. For one and a half months, I make better money than what I make in the village in a year” (Kalli, IDI 12)

However, not all participants agreed with the anticipated benefits of income and remittances during migration. Many believe that these projected benefits of migration are overstated and do not reflect the actual benefits or advantages as anticipated by most migrating households. They contend that the income earned during migration is often insufficient for savings or investment in capital projects or assets.

“Even though I earn well (during migration), it is just better than what I did (earn) in the village. Money earned during migration is never enough to buy assets, build a house, buy a car, etc. it just serves the purpose of immediate family care. If I go to work outside, I earn money to care for the family, but if I do not go outside to work, then I am not able to take care of my family and will just be that empty here in the village and not be able to do anything. I feel migration is a good thing for me. It is improving my family's well-being,” (Vikram, IDI 7)

The benefits of migration tend to increase with distance, and when more individuals from a single household migrate. In such cases, the cumulative remittances or savings from all migrating household members can appear substantial or adequate for that household. Often, entire households consisting of three to five members migrate, utilizing the monthly wages of two to three members for sustenance and expenses, and securing a better monetary saving.

### 4.3 Usage of remittance and income from migration

Participants were reluctant to respond about their income and remittances earned from migration. However, when inquired about the purposes for which their savings and remittances were allocated, participants were more forthcoming and willing to discuss how these funds are put to use. Multiple participants provided similar responses, that mention large expenditures such as repaying loans, expenditures made towards housing, agriculture, health issues, and marriage or festivals.

“When I send money to the family, the money is used for housing purposes, agriculture (farm) work, and treatment of a family member that is sick” (Ranjit, IDI 9)

### 4.4 Effects of migration conditions on health of the migrants

#### 4.4.1 Lack of adequate housing

The availability of suitable housing is instrumental in meeting basic needs, providing rest and relaxation, ensuring safety, and hence promoting physical and mental health. Conversely, the absence of proper housing can result in sleep deprivation, exhaustion, and fatigue, ultimately leading to diminished performance and productivity. Our analysis reveals that participants who lack access to adequate housing often experience stress and sleep disturbances, which affects their overall well-being.

“When we arrive at the place where the job has been secured for us, we do not get any accommodation; we stay in the open area. The employer provides us with tarpaulins. We cut or use wooden sticks to erect a structure and cover the roof with a tarpaulin. Sometimes we get zinc sheets to use as roofs for the temporary structure. We do not have a door for the shed, so it is always open.” (Ajay IDI 4)

"When we migrate during the rainy season and make a hut in the open field, the rain always floods inside the tent. We also face challenges with mosquitoes, dogs, and cattle entering the hut freely. They damage things inside the hut and eat our food." (Surya, IDI 13)

#### 4.4.2 Absence of water and sanitation facilities

Migrants working in agricultural and plantation fields around urban areas face considerable challenges: the absence of essential sanitation facilities, including access to water, toilets, and bathrooms at their migration destinations. Participants primarily rely on water from open wells and water for irrigation purposes, which exposes them to water-borne diseases.

“In the agricultural field, there is always water for irrigating the fields, so we also use this for cooking, drinking, and bathing.” (Tamia, I DI 6)

Another pressing challenge faced by migrants is the lack of adequate sanitation facilities at their destination sites. Participants recounted their difficulties in dealing with this absence, particularly since they often migrate as whole households, including women and children. The employers do not generally provide these crucial amenities, forcing them to adapt to unhealthy practices.

“At the migration place, we did not have bathrooms, so we used some wooden sticks in the field and covered them with tarpaulin. When a person bathes, you can see the person's legs and shoulders outside. Sometimes we just bathe in the open. It is same for women. They bathe with their clothes and change to new clothing inside the hut.” (Amitabh, IDI 10)

“We defecate in the open. We do it before sunrise or in the evening when it is still dark. In emergency conditions during daytime, we either go to a forest or in an uncultivated part of the field near the edge. Since, we are many, yet it is difficult but have to manage in the same spot” (Idi, IDI 11)

#### 4.4.3 Work-related conditions

Participants in construction jobs highlighted that they regularly handle heavy loads such as sand, gravel, and cement, which leaves them physically exhausted and tired every day. Additionally, some participants noted that they work in dusty environments without access to Personal Protective Equipment (PPEs). Most of the migrant labourers complained about body ache and respiratory illness. Plantation workers are also faces chemical exposures without any precautions.

“When planting is complete, also engage in chemicals/ herbicide application. We use the nasal sprayer that we hang on our back, spraying the field through the valve while pumping with one hand. We are not given any PPEs and we often come in contact with herbicide and get a bath on and soaked in it when the wind blows it back to us.” (Amitabh, IDI 10)

#### 4.4.4 Absence of health facilities

The results of this study underscore the healthcare access challenges faced by migrants throughout the migration cycle. In general, the residences of migrant labourers working in agricultural plantations, are provided at a considerable distance from the fields. These places lack healthcare facilities. This geographical separation poses a substantial barrier to migrant households in need of healthcare services when they fall ill. Participants also mentioned that, even in urban locations, they often remain unaware of availability of affordable healthcare facilities.in nearby areas. Furthermore, participants pointed out that healthcare services in urban areas can be prohibitively expensive, with migrants having to bear the full cost themselves. Consequently, many are compelled to return to their place of origin for more affordable medical treatment.

“When I got sick during the last migration, I had to go to government hospitals in a nearby town for treatment. I paid all the expenses as our employer did not cover treatment expenses. Medical expenses are high in cities. I cannot afford to stay longer at the hospital. I had no choice but to come back to my village for treatment.” (Kau, IDI 14)

Another noteworthy problem arising from the limited access to healthcare services is the lack of immunization and vaccination for migrating children, leaving children without necessary immunizations. Participant disclosed that since parents and even nursing mothers have long work hours. They cannot make time to take their children out for healthcare services.

“When I am with my wife at migration place, we work from 7am- 5pm with only one hour break from 1pm-2pm, there is no time for you to go out and do any other thing.” (Ajay IDI 4)

### 4.5 Effects of migration on children

This section delves into the economic effects of migration on both accompanied and left-behind children, providing an overview of their effects on economic and educational scenarios. It offers insights from the perspectives of migrant children as well as those who are left behind. It presents narratives illustrating the effects of migration on both accompanied and left-behind children, emphasizing the socio-economic roles they play both during the migration process and in their home communities.

#### 4.5.1 Accompanied children

Migration is more common in the studied community, which sometimes include children as well accompanying their parents, particularly among families migrating for short-term labour in construction sites and agricultural plantations originating from the villages. Respondents indicated that independent migration typically commences at the age of fifteen. Within these employment settings, children often miss educational opportunities as parents refrain from sending them to school. Such participants claimed that either there is an absence of schools in their work location or a lack of information about the availability of schools in proximity.

“I went with my boy and girl where I work with my wife. I am unaware of any school there. The children play when we go to work” (Vikram, IDI 7)

However, for permanent or long-term migrants, the process of assimilation and the acceptance of education has been improving over time within their migration cycles. This improvement is primarily attributed to their increased interaction with the native population at their destination. The long-term migrants are trying to ensure at least primary education for their children.

“My children are with me at the migration site. They go to a private school that is about 11 km away from the farm. The school is far away, so I arranged a vehicle that takes them to school and back every day.” (Amitabh, IDI 10)

When we probe why he spends money on his children's education, he narrates his experience thus:

“I have lived there (migration place) for a long time, working as a field supervisor and renting a farm. People send their children to school by hiring motorcycles or vehicles. I feel this is good; if not, they would not be spending so much money. So, even though I do not earn much, I imitated what they do and enroled all three children in school, and maybe their lives will be different tomorrow.” (Amitabh, IDI 10)

Additionally, children who accompany their parents during migration engage in various activities. Children help in harvesting and cultivating crops in cotton and paddy sites. Meanwhile, children at construction sites assist their parents in tasks such as brick drying and relocation for construction purposes. Sometimes children do not assist their parents in the field in order to take care of their younger siblings while their parents work.

“Some employers allow the child to partially work for less pay. Sometimes, when this is not possible, they help their mothers on plantations. Some parents encourage their children to work” (Vikram, IDI 7)

#### 4.5.2 Left-behind children

Non-migrating children, whose one or both parents are migrating, generally remain behind under the care of their grandparents. Girls, in particular, take on household responsibilities that include cooking, cleaning, tending to livestock, assisting with cultivation, and preparing meals for the household. Young adolescent and teenage boys engage in farming activities, gathering wide fruits and firewood for the household.

“I stay with my grandparents here in the village. My parents migrated to work outside, and I stayed back to continue with my education. My grandmother brings vegetables from market and I cook. I enjoy it cooking for them.” (Kashvi, IDI, sch. Girl, age 17)

Our adolescent respondents believe that their daily contributions to household chores contribute to the well-being of the household, and they have opportunities to interact with their peers at their own place.

“I take care of livestock when my grandparents go to work in the field. I feel good as I am helping the family grow. During these times in the field, I have the freedom to interact with and play with friends. Even at home, I can go out and meet with a friend when I have finished cooking.” (Aja, IDI. school girl, age17)

Yet, extensive and prolonged engagement in tedious work has detrimental effects on teenage and adolescent children, who report being tired and stressed, especially during intensive paddy (rice) cultivation and harvesting periods.

### 4.6 Effects of migration on education: schoolteachers’ perspectives

This section focuses on the effects of migration on children's education at their place of origin, as viewed by primary school teachers. We provide an overview of the various facets of migration's influence on education, covering school enrolment, attendance, academic performance, parents' perspectives, and the supportive role of teachers in the education of children at their place of origin.

#### 4.6.1 School enrolment and attendance

The school administration insists on enroling students in school and ensuring consistent attendance toward achieving universal primary education. Even if children get enrolled in primary schools, since many children aged 7–10 years migrate with their parents to take care of their younger siblings, there is a high drop-out rate within the community before the completion of primary schooling. Conversely, those who remain behind are consistently engaged in household chores, which hinders their regular school attendance.

“We do not know when the children migrate; they never inform us that they will migrate with their parents. We only know that they have migrated when we stop seeing them in school after 2–3 days. If they miss school for 2–3 days, we assume they are helping their parents in the house, but when it is >3 days, we know that they have migrated, and we may not see them for maybe 2–3 months before they come back. We know that they have returned to the village when we see them back in school” (Charan, KII, school teacher)

“We have at least 8–10 out of 54 children dropping out each week.” (Charan, KII, school teacher)

Our findings also suggest that the rate of dropout does not vary by gender, but the reasons are certainly different. Females drop out earlier than males. Upon closer investigation, it was revealed that as students advance from lower standards to higher standards, the decline in dropout rates becomes more pronounced among girls. Female students discontinue schools to take care of siblings and other household chores, or even pertaining to marriage. While, males drop-out looking to economically help their families.

“Most students drop out when they reach 15–20 years. Girls are married at early age, and boys migrate out of the district at their school completion age.” (Dipak, KII, sch. teacher)

#### 4.6.2 Academic performance and graduation rate

The duration for which migrant children remain absent from school varies among families and is dependent on the family's migration duration. Our findings highlight that the continual absence of children from school has an influence on their school performance and passing rates at higher classes, as they miss classes and crucial assessments. Sometimes students do not come back to schools after they return from migration.

“Because students miss many weeks or even months of classes due to migration, it becomes difficult for them to catch up after they return. Sometimes they miss the period of registration for the standard-5 examination. When the parents migrate, in some cases children do not come to school”. (Charan, KII, sch. teacher)

#### 4.6.3 School attendance and family socioeconomic status

Children from families with poorer socioeconomic status drop out at a higher rate as compared to the well-off households. Since the better-off families are less likely to engage in frequent and long-term migration disrupting children's academic progress. In such cases, typically, only the father migrates, while the mother remains with the children, ensuring continued school attendance.

"When a family is in financial distress, children of that household also stop going to school, or even migrate to earn for the family” (Charan, KII, sch. teacher)

In certain cases, when parents earn better in place of migration, they tend to enrol their children in schools at their migration destination. The teachers noted that while this is very rare, parents who place a high value on education do take such measures.

“When a parent migrates to another place, they take their children with them, and their education stops. If they are getting more money from the migration site or they are educated, they send their children to school in that place; otherwise, the children will work with them on the site. If the child is up to 12 years old, then they help their parents at the site” (Panchayat Leader 2)

#### 4.6.4 Parental support and perception of education

From the teachers' perspective, it is evident that many parents in the communities are not fully aware of the importance of education. As a result, they do not give considerable attention to their children's school attendance, nor do they actively monitor their progress. When left to their own devices, many children express a desire to follow in their parent's footstep as farmers. One teacher shared the following insight during the interview:

“Earlier, parents were not careful about their ward's education and were unaware of readmission process after they return from long-term migration, leading to a higher drop-out rate. But now they prioritize education, have learned the process of readmission. They send their children back to school, even if they stay at the migration place for over 6 months.” (Dipak, KII sch. teacher)

“When workload is high, parents stop their children from going to school to help with the farm work, until the farm work is complete.” (Charan, KII, sch. teacher)

Furthermore, the teachers revealed that parents frequently take their children on migration journeys during the school year, and the children only return home during festive periods in March and April. These are the months when primary schools are on holiday. Importance of education among this community has traditionally been very low. However, scenarios are improving as majority of children eventually return to school, which was less likely a decade back.

#### 4.6.5 teachers as tutors and support aids

4.6.5

Teachers often assume roles as mentors, and mediators to motivate and facilitate children's school attendance. The school principals and teachers from all three primary schools, reported their interventions and meetings with parents, offering guidance to encourage parents to either enrol their children in school or allow their children to attend school.

“We hold meetings with the parents and the children separately to encourage them to attend classes, but many students are not interested in studies. They just follow their parents to migration. Those that do not migrate follow their family members to the farm or take animals for grazing.” (Dipak, KII, school principal)

“When children come back from migration, we counsel the parents and the children, going to each of their houses to send their children back to school, but its not being effective.” (Akhilesh, KII, school teacher)

The school principal discussed the challenges faced by returned migrant children in catching up with their academic work after an extended absence from school. In such cases, teachers also offer extra support and encouragement to help these students stay in school.

“Many of them are not able to write; they do not know the answers. We provide answers to them. Only 10 % have these issues, and we work with their parents to help them graduate from primary school proceeding to standard-6.” (Charan, KII, sch. teacher)

## 5 Discussion

Our study is among the first in India to collectively investigate tribal migrants' perspectives on health conditions at urban migration destinations and teachers' perspectives on the effects of migration on the education of children at their places of origin. This study highlights that even if migration decisions are made to ensure betterment in household economic condition among a rural tribal community in India, the migration conditions adversely affects health of migrants and their accompanying family members due to lack of basic health amenities at their destination. The education status of children of migrating families is also found to be concerning pertaining to high drop-out rate.

The results underscore the motivation of migration as a livelihood strategy (De Haan, 2002; Bahinipati et al., 2021; Carte et al., 2019). A strong association between livelihood vulnerability and multiple-member household migration observed in this community. Share of households with at least 1–2 migrating individuals at each period of the year has risen to an average of 4–5 or more in households based on household size (Attah-Otu et al., 2024). Lower-income households tend to have higher number of migrating members within the same communities in India. However, our analysis further indicates that migrating households feel their migration households have better socioeconomic status when compared with non-migrating households, with remittances used mainly to meet household needs such as food, clothes, and settling accumulated debts. This findings align with an earlier research work (Davis and Lopez-Carr, 2014) that records remittance usage to meet immediate household needs. However, some participants remain skeptical about the long-term effects of migration on alleviating household inequalities or poverty eradication.

Findings indicate that migrants have difficulties accessing essential housing and sanitation needs and are forced to manage with makeshift accommodation and sanitation facilities (Devkota et al., 2021), increasing the risk of vector-borne diseases. These findings aligned with the results of previous studies in India (Pardhi et al., 2020; Srivastava and Sutradhar, 2016) and in China (Swider, 2015) which also recorded poor housing and sanitation facilities among the migrant population. The inability of migrant households to access healthcare services poses a big obstacle where, in most cases, they are forced to return to their community for healthcare services, especially pregnant and lactating mothers who are forced to travel long distances to reach their community for health care, with reduced antenatal care and immunisation recorded in numerous studies conducted in India (Ravindranath et al., 2019). Children at migration destinations are not excluded from this precarious and vulnerable situation as they also cope and adapt in the same way as their adult parents. The persistent movement of parents from one agricultural plantation location to another hinders the children's chances of getting immunisation and vaccination. Furthermore, migrant mothers are constantly busy with their work schedules and do not have time to take care of healthcare needs of their children. This also aligns with previous studies that reported a lack of healthcare services such as nutrition, vaccination, and immunisation for migrant children in India (Kusuma et al., 2010; Vikram, 2023; Raviindranath et al. 2019), and in Ethiopia (Schott et al., 2013; Nguyen, 2016).

Findings further revealed that migrant children are engaged in numerous economic activities that range from being engaged in petty short-term labour work at the construction site, such as carrying or drying bricks, to assisting the parents in agricultural operations such as cultivation and harvesting while also serving as caregivers to their younger siblings in the absence of their parents. These findings correspond with previous studies (Rajan et al., 2023; Srivastava and Sutradhar, 2016) that recorded migrant children workforces in construction industries in India.

However, the engagement of children in minor short-term labour work and the lack of school facilities compromise the education of migrant children. Most parents lack awareness of the availability of educational facilities within their migration destination. This can be attributed to the level of education and exposure of the migrant household. It has been observed that long-term migrants adapt to the conditions much better, and in most cases since their families accompany them, they demonstrate higher levels of awareness of the need for education.

Parent out-migration and leaving children behind with grandparents is a common trend among rural migrants especially in areas with high mobility rates (Abdi, 2018; Singh, 2020). Our study reveals that in the absence of the parents, left-behind children take up different household economic activities to support their grandparents or guardians. There is a gender-wise variation, while girls predominantly take care of household chores, boys contribute more in physically challenging activities, and economic well-being. Since, non-migrating children and adolescents engage in household duties, agricultural works, livestock grazing for long hours, they feel bored and exhausted. Most likely, they find such activities as opportunities to meet, play, and interact with peers. These findings are in agreement with the findings of a systematic review study (Fellmeth et al., 2018) that documented loneliness, anxiety, and depression among children in the absence of their parents. The young non-migrating members showed strong adaptability and resilience. However, they were avoiding schools in absence of their migrating parents. This result is in alignment with previous findings on left-behind children documenting extensive stress among left-behind children (Ai et al., 2016; Huang et al., 2015), challenged in educational performance and attainment (Antia, et al., 2022). While trying to understand association of migration with school dropout and educational attainment, we observed that migration results in low school enrolment, attendance, and achievement, as well as frequent dropout among students irrespective of whether they migrated with their parents or were taking care of family responsibilities in the village itself. This finding is consistent with previous studies (Das et al., 2020; Jampaklay, 2006; Roy et al., 2015; Vikram 2018) that indicate low school enrolment, attendance, and performance among migrating children, as they find it tough to catch up with as they have missed critical lessons and evaluations.

The discussions with school teachers revealed that the drop in girls' attendance is higher as they advance to higher standards pertaining to marriage and family's economic hardship (Coffey, 2013; Vikram, 2018). Students from well-off households do not accompany their migrating parents, and show more academic consistency irrespective of their parent's migration status.

Parental awareness and interest in education also play a crucial role in the academic achievement of children. On the other hand, grandparents and guardians of left-behind children do not prioritise school attendance. Individuals from lower socioeconomic background tend to prioritise household work such as farming and taking care of livestock over the education of their children. This is attributed to low awareness of the necessity of education among tribal communities and even regular counselling from the teachers do not help the scenario. This calls for more policy intervention from the government.

In rural India, the patriarchal system, caste, cultural and gender norm influences and shape the migration decisions and processes within households, where decisions are made by the family head, and every other member of the family is bound by norms formulated by the household heads. The size and composition of the migrating household also influences migration decisions, with households having more members, especially young males, usually have more people migrating. Likewise, gender norms also influence household migration scenarios. Males migrate more frequently, while female require approval from parents or guardians and must migrate and work in the company of a male relative.

## 6 Study strengths and limitation

### 6.1 Strengths

This study offers substantial contribution to the existing body of knowledge. This is well-known that in poor communities rural to urban migration decisions are made to tackle economic vulnerability. However, this unique study has captured its influence on health and educational conditions among migrant families. Secondly, our study offers a comprehensive coverage of multiple aspect of such as motivation, perception of migration, conditions place of origin and destination. Thirdly, this study highlights need for policy measures to provide migrants with health facilities at their place of work and improvement of educational attainment among migrating rural communities. Finally, our study findings highlight that even after serious efforts by the policymakers, early-age marriage and child labour are still prevailing in India. This study results are not only relevant in only tribal or Indian context but also for other countries facing rural to urban labour migration to mitigate financial vulnerabilities.

### 6.2 Limitations

The findings of this study need to be evaluated through the lens of a few limitations. Firstly, language barrier and reliance on a translator slowed down the interview process. We needed to validate the transcriptions and observations with community members and experts. Identifying and interviewing the participants had been a difficult task, and in cases we needed to discuss with an individual more than once. Given the unique social and cultural context among tribal communities, this study has adopted a qualitative approach; and hence the results may not be generalizable.

## 7 Policy implication and consideration

The findings have great implications for policy development and intervention. Provision of good residential, water and sanitation facilities is mandatory to improve migrants’ health condition. Primary health facilities supporting maternal and child health needs, mobile health camps and ambulances should be made available in residential slums of migrants to ensure timely access to healthcare. Secondly, strengthening and enhancing existing rural livelihood development policies such as the Mahatma Gandhi National Rural Employment Guarantee Scheme (MGNREGS) and other employment generation schemes, especially in highly mobile rural tribal areas. At the place of origin, mass awareness and advocacy campaigns including media should be conducted to emphasize the benefit of education and encourage school attendance. Scholarships, and vocational education can result in better academic outcome, boast income generation and reduce livelihood vulnerability. Thirdly establishment of public-funded primary school in urban slum for the migrant children, providing mid-day meal facilities can improve educational continuity among migrant children.

## 8 Conclusion

Our study presents a comprehensive analysis of the multifaceted and complex dynamics of labour migration among rural tribal communities in India. The findings provide concrete evidence of the detrimental effects of migration on health of migrants and education of their children, highlighting both immediate and long-term implication for the well-being of migrants households and their community. This study identifies despite migration plays a crucial role to the economic development of migrating families; it also has adverse effects. The health and educational vulnerabilities are prevalent due to lack of policy focus on migrating communities in origin and destination, as well as the desperation of the households due to economic distress.

Through this study, we presented crucial insights to alert and inform policy implication for government and private sector partnership. The researchers hope that this exploration, analysis, insights, and discussion provide deeper understanding of the migration dynamics and its implications on health and education among under-privileged rural households.

## Data availability

Due to the sensitive nature of the topic and the anonymity of participants, data for this study are not for sharing.

## CRediT authorship contribution statement

**Bernard Attah-Otu:** Writing – review & editing, Writing – original draft, Methodology, Investigation, Formal analysis, Data curation, Conceptualization. **Nikita Jaiswal:** Investigation, Data curation. **Priya Gupta:** Writing – review & editing, Supervision, Methodology. **Angan Sengupta:** Writing – review & editing, Supervision.

## Declaration of competing interest

The authors declare that there is not conflict of interest.
